# Caregiver acceptance of human papillomavirus vaccine for their female children in Chileka, Blantyre, Malawi

**DOI:** 10.1016/j.jvacx.2024.100557

**Published:** 2024-09-18

**Authors:** Akim N. Bwanali, Petro Liundi, Adriano F. Lubanga, Samuel L. Mpinganjira, Luis A. Gadama

**Affiliations:** a Department of Epidemiology and Biostatistics, Kamuzu University of Health Sciences (KUHES), Blantyre, Malawi; b Clinical Research Education and Management Services (CREAMS), Lilongwe, Malawi; c London School of Hygiene and Tropical Medicine (LSHTM), London, United Kingdom; d Department of Obstetrics and Gynaecology, Kamuzu University of Health Sciences (KUHES), Blantyre, Malawi

## Abstract

**Introduction:**

Malawi is one of the countries enduring an enormous burden of cervical cancer. Human Papillomavirus (HPV) vaccination offers a feasible and effective tool for reducing the burden. Such a prospect however, is being impeded by low uptake rates of the HPV vaccine in the country. This study, therefore, sought to identify barriers to caregiver acceptance of the HPV vaccine for their female children in Chileka, Blantyre, Malawi and to establish the consequential willingness to vaccinate their children.

**Methodology:**

A cross-sectional study was conducted using qualitative methods. We conducted 6 Focus Group Discussions (FGDs) with 4 groups of women and 2 groups of men in 4 villages located in a semi-urban area, Chileka, Blantyre. The guiding questionnaire was designed to draw out two main outcomes: barriers to caregiver acceptance of the HPV vaccine and willingness to vaccinate children. The data was analysed by thematic analysis by an inductive approach using NVivo software version 11.

**Results:**

Lack of knowledge on cervical cancer and HPV vaccination coupled with numerous misconceptions are the main barriers discouraging the population from accepting the HPV vaccine. Consequently, there was little desire to get their children vaccinated against HPV. Though responsibility to get the children vaccinated was mainly attributed to women, men argued that they need to have a final say in their children’s vaccination.

**Conclusion:**

In the wake of the revealed barriers and low acceptance of the HPV vaccine, it is imperative to organize effective and sustainable awareness programmes for the improvement of the HPV vaccine’s uptake.

## Highlights

• Cervical cancer poses a major health burden in Malawi.
• Vaccination against the Human Papillomavirus (HPV) offers a feasible tool for reducing the burden.
• Lack of knowledge on cervical cancer and the HPV vaccine and misconceptions hinder the uptake of the HPV vaccine.
• Comprehensive and sustainable awareness programmes should be designed and implemented to address the barriers to HPV vaccination.

## Introduction

Cervical cancer is the fourth most common cancer among women globally, with an estimated half a million new cases and over 300,000 deaths in 2020 [1]. Low and middle-income countries (LMIC) contributed about 90 % of the global deaths due to cervical cancer. This could be attributed to the high prevalence of the Human Immunodeficiency Virus (HIV) pandemic and the lack of effective cervical cancer screening programs in LMIC [2], [3], [4], [5]. Malawi has the highest incidence of cervical cancer in the world, with an age-standardised incidence of 67.9 per 100,000 women and the second highest mortality rate of 51.5 per 100,000 women per year as of 2020 [6], [7]. It is the commonest cancer and the leading cause of cancer-related deaths among women [8]. Alarmingly, over 80 % of Malawian women diagnosed with cervical cancer are at an incurable stage [9].

About 99 % of cervical cancer cases are caused by HPV infection [10], [11]. With an average of 24 %, the sub-Saharan Africa (SSA) bears one of the highest HPV prevalence in the world [12]. High-risk HPV subtypes 16 and 18 together are responsible for about 70 % of cervical cancer cases worldwide [11], [13]. There are numerous risk factors for cervical factors linked to exposure to high-risk HPV subtypes. These include sexual intercourse at a young age (<16 years old), history of sexually transmitted infections, multiple sexual partners and low immunity [14]. Other environmental risk factors include cigarette smoking and low socioeconomic status. These risk factors are prevalent in SSA, hence the region carries the greatest burden of cervical cancer, with 24.55 % of the global mortality from cervical cancer [15]. However, studies done across the SSA have reported that many women are not aware that HIV (which has a high prevalence in SSA), HPV and not being screened for cervical cancer are significant risk factors for cervical cancer [16], [17]. The health belief model alludes that knowledge of a disease and the associated perceived susceptibility, perceived severity, perceived barriers, perceived benefits, and cues to action are imperative for planning prevention and uptake of interventions [18], [19]. It is well-known that community and individual awareness of cancer risk influence risk reduction and help-seeking behaviour [17].

Vaccination against HPV reduces cervical cancer incidence rate by about 90 % [20], [21]. Fittingly, in an effort to curb the global cervical cancer burden, the World Health Organization (WHO) recommends the provision of the HPV vaccine to young girls prior to the onset of sexual activity (before exposure to HPV) [22], [23]. This is a primary target group for routine vaccination (defined by many countries as ages 9–14 years) [24]. Two doses were initially recommended to be administered 6 months apart. Recently, the WHO has recommended that one dose of the HPV vaccine provides adequate protection [25]. Currently, three types of the HPV vaccines are available: the nonavalent Gardasil-9 (protects against genital wart-causing HPV types 6 and 11, and against high-risk HPV genotypes 16, 18, 31, 33, 45, 52), the quadrivalent Gardasil (prevents infections from four HPV genotypes, 6, 11,16, and 18) and the bivalent Cervarix (prevents infections from HPV genotypes 16 and 18) [26].

Many countries administer the HPV vaccine to young girls according to the WHO recommendation. Getting the population to accept and permit girls to get vaccinated is key to meeting the WHO target of fully vaccinating girls by 15 years of age by the year 2030 [23], [24]. Despite the common occurrence of insufficient information about the HPV vaccine, a few studies have reported high acceptance of the HPV vaccine [27], [28], [29]. However, studies done across some sub-Saharan Africa (SSA) countries revealed that common pullbacks to vaccination against HPV include uncertainties over the vaccine’s safety profile, the quality of delivery, the impact on girls’ fertility and other adverse effects [27], [28], [29], [30].

Malawi rolled out a nationwide vaccination programme in January 2019 [31]. This followed an HPV vaccine pilot programme in Rumphi and Zomba districts, which was done in 2013 [32]. In the pilot programme, the quadrivalent Gardasil vaccine was given to Standard 4 pupils and out-of-school adolescent girls aged 9–13 years at 0 and 6 months according to WHO recommendations [24]. In Zomba district, the overall coverage for the first three years was 82.7 %, and the dropout rate was 7.7 % [32]. In Rumphi district, overall coverage was 91.3 %, and the dropout rate was 4.9 %. Vaccine refusal was one of the main reasons for partial or no uptake of the vaccine.

Since the incorporation of HPV vaccination into the routine Expanded Program on Immunisation (EPI) in January 2019, there is emerging evidence that vaccine hesitancy is negatively influencing the vaccine’s uptake in Malawi [33]. For instance, administrative data of the year 2023 reveals an underwhelming 28 % HPV vaccine coverage despite the vaccine being available in the country [34]. On the contrary, the neighbouring country, Zambia reported a coverage of 58 % in the same year, 2023. Vaccine hesitancy drivers linked to routine childhood immunisation have also been attributed to HPV vaccine hesitancy [35]. These include inadequate awareness of the vaccination schedule, rumours and conspiracy theories exacerbated by religious beliefs, and low literacy levels of caregivers [35], [36]. However, there have been few qualitative studies specifically examining the community’s perception toward HPV vaccination. Furthermore, there have been prevalent social media rumours claiming that the program is a conspiracy aimed at impairing young girls’ reproductive health to render them infertile and control population growth. Such rumours have potentially contributed to massive declines in the vaccination’s acceptability, as evidenced by the fluctuating nationwide coverage rates for the HPV vaccine, from 72 % in 2020, to 14 % in 2022 and 28 % in 2023 [34].

Additionally, in 2020, the Kamuzu University of Health Sciences (KUHES) in collaboration with the John Hopkins research institution run a separate campaign to increase the uptake of HPV vaccines that were on the verge of expiring. This speaks of how much acceptance for the vaccine has declined. Despite the fact that a study done prior to the HPV vaccination campaign elucidated a high acceptance rate of 88 % , it examined women’s perspectives only, yet in African contexts, men are the principal decision-makers at the household level [29], [37]. Furthermore, with new vaccines being introduced in the EPI and fluctuating public interest in new vaccines, acceptance of the HPV vaccine may have changed over time. Therefore, this study aimed at identifying barriers to caregiver (both men and women) acceptance of HPV vaccine for their female children in Chileka, Blantyre, Malawi and establishing the consequential willingness to vaccinate their children.

## Materials and methods

### Study design and setting

This was a cross-sectional qualitative study. It was conducted in Chileka, a semi-urban area located 13 km, South-west of the commercial city of Blantyre, in the Southern Region of Malawi. The study involved 4 local villages, namely, Golowa, Dambo, Singano and Mkunthiwa. Blantyre District was conveniently chosen since the principal investigator was studying at the KUHES during the study period, which is located in the city, making Blantyre an easily accessible area.

### Study population and sampling

The study participants were caregivers (both married and single) with at least 1 female child within the age range of 9–14 years, regardless of the vaccination status of their child. Caregivers whose 9–14-year-old female children had not been staying with them for at least 6 consecutive months were excluded from the study. We supposed the decision to get the children vaccinated would primarily lean on the caregiver staying with the children.

A multistage sampling strategy was employed. Firstly, Blantyre was conveniently chosen. Secondly, a convenience sampling method was used to choose 4 easier-to-reach semi-urban villages in the semi-urban Chileka area as the exact study locations. Finally, the participants within the villages were purposively selected (those with at least 1 female child aged between 9 and 14 years).

### Data collection

Data was collected using focus group discussions (FGDs). There were separate FGDs for women and men, since in Malawian cultural context, women frequently find it difficult to express themselves freely when around men. A total of 6 FGDs were conducted, with 4 FGDS with women in each of the following villages: Golowa, Dambo, Singano, and Mkunthiwa, and 2 FGDS with males in Golowa and Singano villages. The FGDs began with obtaining the participants’ informed consent, and were conducted in their preferred language (the vernacular language, Chichewa). Two research assistants who were trained and experienced in qualitative methods facilitated the FGDs. The questionnaire that guided the FGDs was designed to specifically elucidate two main outcomes namely, barriers to caregiver acceptance of the HPV vaccine and the associated willingness to get children vaccinated. A single session ran for 45 min to an hour and was recorded on sound recorders, after obtaining the participants’ permission. Key qualitative interview characteristics such as, moderating research assistant, FGD date, site, language, setting and duration, respondent characteristics and audio file information were listed in an Excel spreadsheet.

### Data management

The audio recordings were transcribed verbatim within 72 hours by the FGD facilitators. The transcripts were then translated into English language. To ensure confidentiality, all electronic records were stored in a password-secured computer and all paper records were kept in a locked filing cabinet. Data was also backed up on Google Drive, and the link was only accessible to key personnel. This was done to prevent data loss.

### Data analysis

The electronic data transcripts were then imported into NVivo software version 11 for thematic analysis using an inductive approach. Common themes or patterns were generated from the data set to develop theories. This was done in six steps according to Clarke and Braun’s thematic analysis [38], [39]. Firstly, the research team members familiarised themselves with the data set. Then, they generated data codes representing specific basic segments of the data. This was followed by grouping codes denoting similar concepts into succinct themes. The developed themes were then reviewed to ensure they fit with the original data set, after which they were clearly defined and named. Finally, a report of the data analysis was generated. The final interpretations were supported by quotes from the FGDs.

### Ethical considerations

The study was approved by the KUHES Research and Ethics Committee. Permission to conduct the study within Blantyre district was sought and obtained from the Blantyre District Health Office (DHO). At the study sites, village heads provided permission to carry out the study in their respective villages. Verbal informed consent was obtained from the potential participants after fully explaining to them the nature and possible consequences of the study.

## Results

### Demographic profile

The participants consisted of 34 women and 16 men. The discussion was done in Chichewa language and all the participants were fluent Chichewa speakers. Participants’ education level ranged from no basic education to secondary education, with an average of 5 years in school. Like most Malawian households, they make a living by subsistence farming and small-scale businesses. All of them had at least 1 child within the age range 9–14 years. Their marital statuses varied but most of them were married and living together with their partners. The socio-demographic characteristics of the study participants have been summarized in Table 1 below.

**Table 1:** Socio-demographic characteristics of the study participants.

**Variable**		**Number, N=50 (%)**
Site	Golowa	16 (32.0 %)
	Dambo	9 (18.0 %)
	Singano	17 (34.0 %)
	Mkunthiwa	8 (16.0 %)
Sex	Male	16 (32.0 %)
	Female	34 (68.0 %)
Age group	<20 years	2 (4.0 %)
	20–30 years	27 (54.0 %)
	31–40 years	15 (30.0 %)
	>40 years	6 (12.0 %)
Level of education	Never attended school	6 (12.0 %)
	Primary education	33 (66.0 %
	Secondary education	11 (22.0 %)
	Tertiary education	0 (0.0 %)
Occupation	Subsistence farmer	29 (58.0 %)
	Small-scale businesses	11 (22.0 %)
	Regular job or piecework	10 (20.0 %)
Marital status	Married	41 (82.0 %)
	Not married	9 (18.0 %)
Source of information on HPV and vaccine	Health facility	19 (38.0 %)
	Local radio stations or television	9 (18.0 %)
	Peers	6 (12.0 %)
	Health education vans	12 (24.0 %)
	More than 1 of the above	4 (8.0 %)
Vaccinated children against HPV	Never	49 (98.0 %)
	At least 1 child	1 (2.0 %)

### Themes

From the analyses of the focus group transcripts, themes emerged regarding knowledge about cervical cancer and its vaccine, misconceptions regarding the HPV vaccine, their willingness to get their children vaccinated and primary responsibility to get the children vaccinated. These main themes are subsequently discussed in greater detail below along with some subthemes.

#### Theme 1: Awareness of cervical cancer and HPV vaccine

##### Knowledge of cervical cancer

Most of the participants had some knowledge about cervical cancer. However, the knowledge was either insufficient or not entirely correct. The women were more confident with what they knew about cervical cancer than the men.

“*Sometimes, cervical cancer comes on its own, sometimes due to sleeping with a lot of men. But if they have found you with it in the early stages, it is curable. However, if it is in the later stages, then there is nothing that the doctors can do.*” (Woman001)

*“Some of us are not aware of how dangerous it is. We have not seen anyone suffering from it. We do not know who gets it, what causes it, its signs and symptoms. We really do not know a lot about it.”* (Man001)

Only one woman stated having unprotected sexual intercourse with an uncircumcised male as a risk factor for cervical cancer.

“*The causative germ originates from uncircumcised men. It is passed on to women during unprotected sexual intercourse*.” (Woman003)

However, none specifically mentioned or knew about HPV, nor the causal link between HPV and cervical cancer.

Only one woman stated encouraging their sexual partners to get circumcised as one of the preventive measures against cervical cancer. Across all the FGDs with women, two respondents mentioned frequent screening at the health centre. They explained that screening diagnoses the disease early enough to be cured. However, none of them promptly mentioned vaccination against HPV as another preventive measure.

*“We need to get tested so we get treated early before the disease spreads. I got tested and was advised to return after 3 years for another test.”* (Woman009)

##### Knowledge of the HPV vaccine

Majority of the women acknowledged that they are aware of the presence of the vaccine provided to protect against cervical cancer in Malawi. (Worth noting that none of them had mentioned HPV vaccination out of their own accord as a preventive measure for cervical cancer.) This knowledge was afforded to them during Parents-Teachers Association (PTA) meeting in the year 2018 where they were notified that their daughters would be vaccinated at their primary schools.

“*I learnt about it from a PTA meeting we had at our school. They said any child who is ready can receive the vaccine.”* (Woman002)

However, they were not sure if the vaccine was currently being provided anywhere within their area, whether in schools or at the hospital.

*“Some time back, we heard that they will be going in primary schools to provide the vaccines to the children. But now, we do not know if it is being provided anywhere.”* (Woman010)

With the men, on the contrary, most of them conceded no awareness with regards to the availability of the vaccine.

*“We do not know if there is a vaccine for cervical cancer.”* (Man005)

The women had different views on the eligibility criteria for receiving the HPV vaccine. None of them stated the exact age ranges for the young girls that qualify the girls to receive the vaccine. Some debated between anyone above the age of 12 years and anyone less than 15 years of age.

*“We heard the vaccine was for people aged at least 12 years.”* (Woman011).

*“Some say it is given to girls aged less than 15 years.”* (Woman012).

Some women claimed they thought the vaccine was for women of all ages. For those who stated young girls as the target population, they could not explain why only young girls are targeted or how the vaccine helps to prevent cervical cancer.

The men, however, generally expressed no awareness on the appropriate age ranges eligible to receive the vaccine.

#### Theme 2: Misconceptions regarding the HPV vaccine

There were two major misconceptions pertaining to the HPV vaccine that were expressed by both men and women. The first one that uniformly came out in all the FGDs was that participants either believed or heard that the vaccine was a government’s plot intended at impairing the young girls’ reproductive health and rendering them infertile in the future to manage the fast-growing population.

*“Some people say the vaccination is a government’s scheme to reduce our population. They are targeting these little girls to make them incapable of bearing children when they grow up.”* (Woman012)

Another misconception that was mentioned in almost all of the FGDs is that the HPV vaccine was alleged to be the COVID-19 vaccine which is believed to have been developed to kill people. This was also attributed to be the key reason leading to most children absconding classes and running away from their schools on the days the vaccine was communicated to be administered.

*“Many people think that this could be the COVID-19 vaccine. Most of the learners ran away from school on the days the vaccine was to be given.”* (Woman011)

#### Theme 3: Willingness to get their children vaccinated with HPV vaccine

Most respondents expressed little to no desire to get their children vaccinated. They backed up this hesitancy with varying explanations, including that they would accept only when their children are tested and found with the disease. This was the common perception amongst both men and women.

*“I cannot allow my child to receive the vaccine unless they convince me of what kind of vaccine the child is getting and its actual function in the body.”* (Woman013)

A small segment of the participants though, expressed readiness to vaccinate their children against cervical cancer despite having limited knowledge about cervical cancer and the HPV vaccine. They perceived the vaccine to be credible as it was recommended by the government.

*“If the Government endorsed it, then we may just have to believe that it is helpful even without knowing everything about the vaccine”.* (Woman004)

The single participant whose children had already received the vaccine expressed strong conviction that the vaccine was harmless as quoted below:

“*My children did not face any problems after receiving the vaccine. I do not know if some problems might arise in the future. But for now, I can say it is safe. I would encourage fellow parents to encourage their children to receive the vaccine”* (Woman013)

Furthermore, the participants who had witnessed someone suffering from cervical cancer displayed the most desire to get their children vaccinated.

*“I have seen a person suffering from cervical cancer. I would not want my children to suffer that way. Once the vaccine comes, I will take my children to the vaccination centre.”* (Woman014)

The respondents that exhibited reluctance to receive the vaccine stated that they would require clear information to be provided to them explaining the benefits of the vaccine and clearing out the rumours surrounding the vaccine.

*“We deserve to know first what our children are getting and why. No one can just come and say, ‘take this vaccine’.”* (Man010)

Generally, across all the FGDs, there was a general desire for further information to be provided to them to motivate more people to accept the vaccine.

#### Theme 4: Responsibility to get the children vaccinated

Most women cited that the woman holds the principal responsibility to decide whether the child gets vaccinated or not.

*“We, the women, are the ones who realise the dangers of cervical cancer, so we need to take the responsibility. When the child gets sick, we are the ones who take them to the hospital.”* (Woman018)

Men explained that the man, as the head of the family, should have the authority whether to permit or deny vaccination for the child.

*“They are my children. So I would be disappointed if my wife gets them vaccinated without asking for my permission because I am the head of the family.”* (Man013)

At one FGD, the women responded that it should be the child’s responsibility to decide whether to receive the vaccine or not.

*“It is advantageous that the vaccine is not being given to a toddler. A 12-year-old is mature enough to make their own personal decisions without any external influence and pressure.”* (Woman019)

Finally, the FGD facilitators provided the study population with information sensitising them to get their children vaccinated. The study population expressed their comprehension and appreciation for the clear and enlightening knowledge.

*“This discussion has been enlightening and educative. Once the vaccine arrives, we will be on the forefront, encouraging our children to get vaccinated. Hopefully, our fellow parents will also be motivated by seeing us getting our children vaccinated.”* (Woman018)

## Discussion

The study examined cervical cancer and HPV vaccine awareness and barriers to caregiver acceptance of the HPV vaccine for their female children in Chileka, Blantyre, Malawi. There is limited knowledge and awareness of cervical cancer and the HPV vaccine amongst the study population. They had a meagre understanding of the causal relationship that has been reported between HPV and cervical cancer as well as the role of the HPV vaccine in the prevention of the cervical cancer. Additionally, they demonstrated lack of knowledge about other methods of preventing cervical cancer. The limited knowledge and widespread misconceptions consequently, are important barriers that hold back many caregivers from encouraging HPV vaccination for their children.

The study population displayed a general lack of knowledge and misinformation about cervical cancer. Similar studies in SSA have shown that such social predisposing factors as low levels of education and poverty are significantly associated with a lack of awareness of cervical cancer, HPV and the HPV vaccine [40], [41], [42]. Several studies on cervical cancer awareness and health-seeking behaviour in various settings in Malawi have also stipulated extremely limited knowledge of cervical cancer [3], [43], [44]. The same could be expected in this study since the study population had low levels of education and are exposed to misleading reports spread on social media. This highlights a common challenge of insufficient information dissemination and comprehension in African settings, which undermines the fight against cervical cancer, including hindering HPV vaccination uptake. In light of this, it is vital to imminently close the knowledge gap through community awareness campaigns, by utilising means that are already convenient to the study population, especially social media platforms.

Furthermore, the study population displayed limited knowledge about the HPV vaccine as a preventive measure until prompted about the vaccine. This is similar to a study done in the same country in 2013, which established that knowledge of the HPV vaccine was stumpy [29]. The aforementioned study was done before the Malawi Government launched the HPV immunisation campaign. Thus, it would be reasonably expected that by now HPV vaccine awareness should have significantly improved since people had been sensitised about the vaccine before implementation of the nationwide vaccination campaign and the information should be relatively new in their memory. This shows that the campaigns were neither effective nor had sufficient coverage. The finding is worrisome because inadequate awareness of the vaccination schedule and low literacy levels have been reported as vaccine hesitancy drivers for routine immunization as well as HPV vaccination [36]. The low knowledge on the benefits of the vaccine and recommended dosing schedules explains why the vaccines in stock at KUHES and John Hopkins research facilities were on the verge of expiring in the year 2020, moving them to run a separate campaign to increase the vaccine’s uptake. This paints a bad picture on the utilisation of the vaccine in the country, which could impede potential donors from providing the vaccine in supplies that satisfy the actual demand on the ground. This calls for wide dissemination of comprehensive information using multiple strategies, including mass media, health facility education, and involving local faith communities. This can ensure that all community members access the correct information, seek appropriate health assistance and make informed decisions about vaccination. It is also important to make it clear that the Malawi Government incorporated the HPV vaccine into the EPI programme, thus the vaccine can be easily accessed in public healthcare facilities.

This study brought to light numerous misconceptions about the HPV vaccine. The study population suspected that it could be the government’s deliberate efforts to control the population by rendering the girl children infertile in the future and also that it has been combined with the COVID-19 vaccine that is believed to be lethal. The first misconception has been reported in several other studies [27], [45]. As in many African countries, infertility issues amongst Malawian women can have serious psychological and social implications for women, along with being a major factor for marital instability, social pressure and ostracism, stigmatisation and abuse [46]. This reduces the acceptance levels for the uptake of the vaccine. This, hence, proposes that promotional efforts to increase the uptake of the vaccine should include deliberate strategies to neutralise the negative rumours and misinformation about the vaccination.

The caregivers in this study generally expressed reluctance to get their children vaccinated. This is contrary to a finding in a similar study done in 2013 in Chiradzulu which revealed an 88 % acceptance rate for the vaccine despite the fact that many participants had extremely limited knowledge of the vaccine [29]. Such is a common finding in SSA that the vaccine has high acceptance rates and that people are willing to receive the vaccine despite having limited awareness about cervical cancer and the vaccine [28], [40], [41], [47]. This has been attributed to trust in the healthcare workforce and low education levels. The change in our study could be attributed to fading trust in the healthcare workforce which was especially rampant amidst the COVID-19 pandemic. Additionally, most of the aforementioned studies are based in rural settings where the potential impact of social media-propagated conspiracy theories is minimal. The semi-urban setting of this study reveals a potential fertile ground for the rapid spread of conspiracy theories due to increasing exposure to social media. These findings therefore suggest a need to establish trust between the healthcare workforce and the community through community awareness campaigns. Fostering such trust could enable people to readily accept the vaccine and not be easily swept aside by malicious social media rumours and conspiracy theories.

The highest acceptance of the vaccine was observed among caregivers who had a personal account of someone who suffered from cervical cancer. This indicates that they understood their susceptibility to and severity of cervical cancer. Similar studies correlate with this finding that the perception of the severity of HPV-related diseases improves acceptance of the vaccine [27], [28]. Hence, in this study, the low acceptance of the vaccine could also be explained by the limited awareness of the severity of the disease and susceptibility of the population. This, therefore, vindicates the incorporation of health belief model constructs such as perceived severity and benefit, when developing education programmes for the community. During education programmes, people who had first-hand experience with cervical cancer could be asked to volunteer to narrate the severity of the disease to encourage the population to accept the vaccine.

The study revealed contrasting views about the primary responsibility for the vaccination of the children. While the majority of the participants view women as having the sole responsibility, some claimed the responsibility lies with the men and the children themselves. Nonetheless, a different study discovered that the need to attain a husband’s approval increased HPV vaccination intention more than knowledge did [36]. This is in unison with a common finding in similar studies in SSA which reveal that men are the decision-makers in African households [37]. Children usually follow their caregivers’ directives as our study found out that the children absconded from classes on the days the vaccine was communicated to be administered as directed by their caregivers. Therefore, it is imperative that information provision also targets all stakeholders including household heads to ensure the success of vaccination programmes.

Some participants in this study argued that the children are equally capable of making an informed decision on whether to receive the vaccine or not. A study on schoolgirls in Uganda showed that perceived benefits of the vaccine amongst school girls encouraged them to go for vaccination [27]. Though they may not be ethically and legally competent to decide by themselves, the reality is that the children cannot be forced, and it is only ethical that they also accept the vaccine by themselves. Therefore, separate studies targeting schoolgirls would need to be carried out in Malawi to establish the young girls’ perception and possible appropriate interventions to encourage the HPV vaccine’s uptake. Furthermore, it is important to incorporate topics on cervical cancer and HPV vaccination into the school curriculum to empower the girls with the necessary information. They could also help their caregivers attain a better understanding of the HPV vaccine, thereby influencing them to readily permit HPV vaccination for their children.

## Conclusion

The study found that poor awareness of HPV vaccination and rampant misconceptions among the study population impede the uptake of the HPV vaccine. This is so despite community awareness campaigns that the government conducted before the launch of the HPV immunisation programme. Therefore, extensive and sustainable community education programmes need to be put in place to ensure high uptake rates of the HPV vaccine. Such diligent efforts will ultimately lead to the realization of the WHO goal of fully immunising all young girls by the year 2030.

Availability of Data

The data obtained and/analysed during the study is available and can be provided upon a reasonable request made to the corresponding author.

Funding

National Commission for Science and Technology Small Grant Scheme.

## CRediT authorship contribution statement

**Akim N. Bwanali:** Conceptualization, Data curation, Formal analysis, Project administration, Validation, Investigation, Methodology, Formal analysis, Writing – original draft, Writing – review & editing. **Petro Liundi:** Data curation, Formal analysis, Investigation, Methodology, Project administration, Validation, Writing – original draft, Writing – review & editing. **Adriano F. Lubanga:** Conceptualization, Data curation, Formal analysis, Project administration, Writing – original draft, Writing – review & editing. **Samuel L. Mpinganjira:** Conceptualization, Data curation, Formal analysis, Supervision, Validation, Writing – original draft, Writing – review & editing. **Luis A. Gadama:** Conceptualization, Validation, Writing – original draft, Writing – review & editing, Supervision.

## Declaration of competing interest

The authors declare that they have no known competing financial interests or personal relationships that could have appeared to influence the work reported in this paper.

## Data Availability

Data will be made available on request.
